# Differences between colon and rectal cancer in complications, short-term survival and recurrences

**DOI:** 10.1007/s00384-016-2633-3

**Published:** 2016-08-06

**Authors:** Max P.L. van der Sijp, Esther Bastiaannet, Wilma E. Mesker, Lydia G. M. van der Geest, Anne J. Breugom, Willem H. Steup, Andreas W. K. S. Marinelli, Larissa N. L. Tseng, Rob A. E. M. Tollenaar, Cornelis J. H. van de Velde, J. W. T. Dekker

**Affiliations:** 1 Department of Surgery, Leiden University Medical Centre, P.O. Box 9600, 2300 RC, Leiden, the Netherlands; 2 Netherlands Comprehensive Cancer Organisation, P.O. Box 19079, 3501 DB Utrecht, the Netherlands; 3 HAGA hospital, Leyweg 275, 2545 CH Den Haag, the Netherlands; 4 MCH, P.O. Box 432, 2501 CK Den Haag, the Netherlands; 5 Groene Hart hospital, Bleulandweg 10, 2803 HH Gouda, the Netherlands; 6 Department of Surgery, Reinier de Graaf Group, P.O. Box 5011, 2600 GA Delft, the Netherlands

**Keywords:** Colon cancer, Rectal cancer, Short-term mortality, Complications, Recurrence, Failure-to-rescue

## Abstract

**Purpose:**

Many apparent differences exist in aetiology, genetics, anatomy and treatment response between colon cancer (CC) and rectal cancer (RC). This study examines the differences in patient characteristics, prevalence of complications and their effect on short-term survival, long-term survival and the rate of recurrence between RC and CC.

**Methods:**

For all stage II–III CC and RC patients who underwent resection with curative intent (2006–2008) in five hospitals in the Netherlands, occurrence of complications, crude survival, relative survival and recurrence rates were compared.

**Results:**

A total of 767 CC and 272 RC patients underwent resection. Significant differences were found for age, gender, emergency surgery, T-stage and grade. CC patients experienced fewer complications compared to RC (*p* = 0.019), but CC patients had worse short-term mortality rates (1.5 versus 6.7 % for 30-day mortality, *p* = 0.001 and 5.2 versus 9.5 % for 90-day mortality, *p* = 0.032). The adjusted HR (overall survival) for CC patients with complications was 1.57 (1.23–2.01; *p* < 0.001) as compared to patients without complications; for RC, the HR was 1.79 (1.12–2.87; *p* = 0.015). Relative survival analyses showed high excess mortality in the first months after surgery and a sustained, prolonged negative effect on both CC and RC. Complications were associated with a higher recurrence rate for both CC and RC; adjusted analyses showed a trend towards a significant association.

**Conclusion:**

Large differences exist in patient characteristics and clinical outcomes between CC and RC. CC patients have a significantly higher short-term mortality compared to RC patients due to a more severe effect of complications.

## Introduction

Beart et al. (1983) and Bufill et al. (1990) were among the first to show clinical and morphological differences between colon cancer (CC) and rectal cancer (RC) over two decades ago. Many studies have since supported this “two types of CRC’s” hypothesis, consequently resulting in a more definitive separation of the colorectal cancer (CRC) group for scientific research and treatment. However, many clinical consequences are still not fully understood [1].

Accumulating evidence suggests etiological differences between CC and RC. Various studies indicate that high body mass index, low physical activity and dietary parameters such as high intake of beef, pork or lamb, processed meat and alcohol are risk factors for CC but not for RC [2–4]. The prevalence of genetic mutations or mutation patterns and hereditary cancer types seem to vary between CC and RC [2]. For CC, K-RAS mutations are thought to be a common early event in carcinogenesis, but are much rarer in RC. RC has more immunohistochemical expression of p53, which has a yet unexplained positive correlation with the patients’ age and is of independent prognostic value for the disease free survival, in contrast to CC p53 mutations [5]. Furthermore, HNPCC is associated with right-sided CC whilst FAP is associated with left-sided (or distal) CC and RC (also known as left-sided CRC) [1].

Anatomically, the colon and rectum are very different in location, blood supply, drainage and innervation. These differences result in dissimilarities in the invasive growth of the primary tumour as well as surgical approaches and treatment outcomes [6]. They are also grounded for differences in the mechanisms for developing recurrences and metastases [7]. The rectal venous drainage bypasses the liver, which explains a higher frequency of lung and bone metastases in RC. Peritoneal spread is much more frequent in colon cancer, which is due to the location of the colon in the peritoneal cavity whilst the rectum is located in the pelvis. Different organ metastases have varying thymidylate synthase expression levels that result in differences in the chemosensitivity of metastases between RC and CC [7–9]. Postoperative fluorouracil-based (5-FU) chemotherapy is successful for CC (especially proximal/right-sided tumours), but its effect on the (disease free) survival of RC patients is still much debated [9, 10]. Besides the anatomical explanations, the microsatellite instability and CpG island methylator phenotypes being associated with proximal (or right-sided) CC and chromosomal instability with distal CC and RC is of importance for the chemosensitivity [10].

Other differences in the response to treatments includes preoperative radiotherapy, which reduces recurrence and is favourable for the survival of RC stage II and III patients whilst this is less evident in CC, also because of a poorly defined target due to the colon’s mobility, and dose-limiting structures in its proximity [11].

The primary treatment for both CC and RC is surgical resection. Resection for both cancer surgeries is considered to be high-risk surgery, with significant morbidity and mortality. Postoperative mortality rates are about 5 % and complication rates range from 20 to 40 % [12–14]. Postoperative complications (such as anastomotic leakage) are associated with mortality due to their association with local recurrences and a prolonged effect of the impact of the surgery, reflected by the 1-year excess mortality rates [12, 15]. The 1-year excess mortality rates vary largely between CC and RC patients. Gooiker et al. (2012) showed 1-year excess mortality rates of 10.9 % for CC patients versus 4.8 % for RC patients [12].

The identification and separation of CC and RC as different malignancies have already allowed for better-targeted and specified treatment. Increasing our understanding of fundamental differences between CC and RC and their pathogenesis even further enables improvement of the prognostic accuracy, treatments and patient care.

From a clinical perspective, it is important to be aware of differences in patient characteristics and in surgical outcomes between CC and RC patients. The aim of this study is to identify differences between CC and RC with the emphasis on differences in short-term survival, complications and the effect on recurrences.

## Patients and methods

All CC and RC stage II and III patients diagnosed between 2006 and 2008 in the Leiden region of the Netherlands Comprehensive Cancer Organisation (IKNL) who received curative surgery were selected from the Netherlands Cancer Registry (NCR). Data managers collected data from the original patient files in one academic and four teaching hospitals: Leiden University Medical Centre (LUMC), HAGA hospital, Medical Centre Haaglanden (MCH), Reinier de Graaf Group (RdGG) and Groene Hart Hospital (GHZ). The data collected included information on the diagnosis, staging, surgery, complications (such as ileus, anastomotic leakage and cardiac or respiratory events), patient characteristics, comorbidities, follow-up time and recurrences. Exclusion criteria included previous colorectal tumours in the patient’s history (*n* = 6), incorrect staging or incomplete TNM-scores (*n* = 52) and missing data on recurrences or the survival parameters (*n* = 13). CC was defined as malignancies located from caecum to sigmoid and RC was defined as malignancies located in the rectum, a tumour within 15 cm from the anal verge. All patients with rectosigmoid tumours were also excluded (*n* = 144).

### Statistical analysis

All statistical analyses were performed using IBM SPSS statistics software version 20.0 and STATA version 12.0. The significance of differences in the patient characteristics was calculated using the Chi-squared test. Relationships between complications and the overall survival (OS) as well as the disease free period (DFP) were assessed for CC and RC patients. All time-to-event variables were calculated from date of surgery. An event for the OS was defined as death due to any cause; for the DFP, any recurrence (locoregional and distant) was defined as an event. Univariate and multivariable Cox proportional Hazard models were used to model the impact of complications on the OS and DFP with adjustments for age, stage, grade and emergency surgery. Short-term mortality was calculated as a percentage of mortality due to any cause. Expected mortality was based on the matched (age, sex, year) general population. Relative survival was calculated as the ratio of the survival observed among the cancer patients and the survival that would have been expected based on the corresponding, age, sex and year-matched, general population. National life tables were used to estimate expected survival (Ederer II method). Relative excess risks of death (RER) were calculated for the differences between colon and rectal cancer using a multivariable generalized linear model with a Poisson distribution, based on collapsed relative survival data, using exact survival times. A *p* value below 0.05 was considered statistically significant.

## Results

Between 2006 and 2008, 767 CC patients and 272 RC patients with stage I, II and III disease were operated with curative intent. Surgery was performed using conventional laparoscopic or laparotomic resection techniques. Pre and postoperative radio-/chemotherapy was administered to qualifying RC and CC patients conform the Dutch “Guidelines of Oncological Health” [16]. The median age of these patients was 72 years (range 25–96 years) for colon and 69 years (range 30–94 years) for rectal cancer. Patient characteristics are shown in Table 1. Significant differences between colon and rectum patients were observed in most characteristics. With respect to patient characteristics, more male patients had rectal cancer (47.8 % for colon versus 58.8 % for rectal cancer). Of all CC patients, 45.4 % was above the age of 75, versus 31.6 % of the RC patients. Comorbidities were significantly more common in CC patients (76.3 versus 68.8 % in RC patients) and emergency surgeries were also more common in CC patients (14.0 %) than RC patients (0.7 %). In addition, differences in T-stage, grade and pre/postoperative adjuvant treatments were observed.

**Table 1:** Patient characteristic, according to colon or rectal cancer

Characteristics		Colon, *N* = 767 (73.8 %)	Rectum, *N* = 272 (26.2 %)	*P* value
Gender	Male	367 (47.8)	160 (58.8)	0.002
	Female	400 (52.2)	112 (41.2)	
Age	<65	206 (26.9)	92 (33.8)	<0.001
	65–75	213 (27.8)	94 (34.6)	
	>75	348 (45.4)	86 (31.6)	
Comorbidity	Yes	585 (76.3)	187 (68.8)	0.015
	No	182 (23.7)	85 (31.2)	
Number of comorbidities	None	85 (31.3)	182 (23.7)	0.04
	1	72 (26.5)	203 (26.5)	
	2	56 (20.6)	159 (20.7)	
	>2	59 (21.7)	223 (29.1)	
ASA score	I	78 (10.2)	34 (12.5)	0.02
	II	228 (29.7)	98 (36.0)	
	III	102 (13.3)	17 (6.3)	
	IV	7 (0.9)	2 (0.7)	
	V	2 (0.3)	1 (0.4)	
	Emergency	7 (0.9)	0 (0.0)	
	Unknown	343 (44.7)	120 (44.1)	
TNM-stage	II	486 (63.4)	178 (65.4)	0.5
	III	281 (36.6)	94 (34.6)	
T-stage	T1	46 (6.0)	18 (6.6)	<0.001
	T2	142 (18.5)	99 (36.4)	
	T3	492 (64.2)	151 (55.5)	
	T4	87 (11.3)	4 (1.5)	
Grade	1	47 (6.1)	5 (1.8)	<0.001
	2	500 (65.2)	123 (45.2)	
	3	112 (14.6)	20 (7.4)	
	Unknown	108 (14.1)	124 (45.6)	
Emergency	Yes	107 (14.0)	2 (0.7)	<0.001
	No	660 (86.0)	270 (99.3)	

### Short-term mortality

As shown in Table 2, there was a difference in the short-term (observed) mortality of 5.2 % (6.7 % for CC and 1.5 % for RC; *p* = 0.001) between CC and RC patients after 30 days. This difference was also evident in the 90-day mortality (with a difference of 4.3 %, *p* = 0.032), but was no longer significant for the 1-year observed mortality (difference of 4.5 %, *p* = 0.075). The proportion of one-year excess mortality is higher for CC (12.5 and 8.5 %), however, not statistically significant (*p* = 0.1). When adjusted for age, T-stage, grade, surgery in emergency setting, comorbidity and number of complications, there was no difference in 1-year mortality between colon and rectal cancer (odds ratio 1.01 (95 % confidence interval (CI) 0.60–1.66); *p* = 0.8).

**Table 2:** Short-term mortality rates and survival for colon and rectal cancer patients

	Rectal cancer	Colon cancer	*p* value
30-day observed mortality^a^	1.5	6.7	*0.001*
90-day observed mortality^a^	5.2	9.5	*0.032*
1-year observed mortality^a^	11.6	16.1	0.075
1-year expected mortality^b^	3.1	3.6	**–**
Excess mortality	8.5	12.5	0.1
Multivariable analyses of the 1-year mortality^c^		OR 1.01 (0.60–1.66)	0.8

The italicized items have a *p* - value smaller than 0.05, which is considered statistically significant in our study

^a^Overall mortality due to any cause

^b^Expected mortality based on the matched (age, sex, year) general population

^c^Adjusted for age, T-stage, grade, emergency setting, comorbidity and number of complications OR odds ratio

### Long-term relative survival and recurrences

**Fig. 1:**
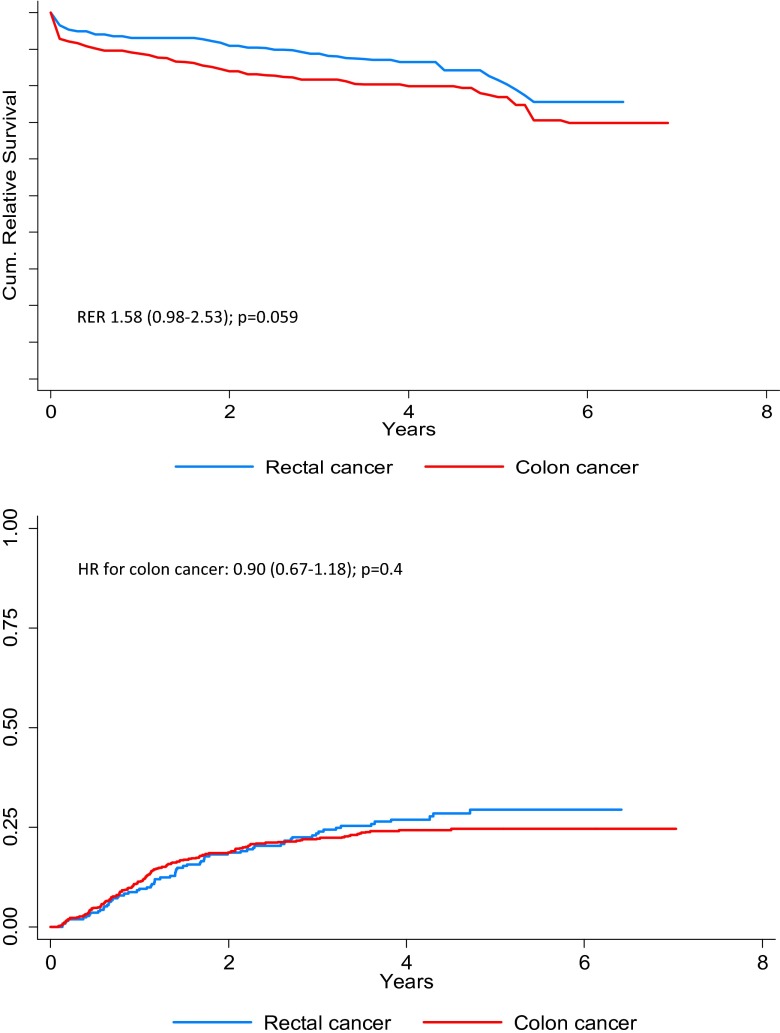
Relative survival (top graph) and cumulative recurrence rate (bottom graph) for colon and rectal cancer patients

The median follow-up time for all patients was 3.5 years (range 0.0–7.0 years). The relative survival (Fig. 1 top graph) for CC shows a steep decline in the cumulative relative survival in the first period after surgery. This decline is absent in the RC patient group. The observed difference in the relative survival between CC and RC yielded a RER of 1.58 (95 % Cl 0.98–2.53); *p* = 0.059. There were no significant differences in the cumulative recurrence rate for CC and RC patients (hazard ratio (HR) 0.90, (95 % Cl 0.67–1.18); *p* = 0.4, Fig. 1 bottom graph).

### Complications

CC patients experienced fewer complications compared to RC patients (41.1 versus 49.3 %; *p* = 0.019). The most common complications were similar for CC and RC: anastomotic leakages, ileus, cardiac events and respiratory tract infections. Of all CC patients, 20.3 % experienced surgical complications and 23.4 % medical complications, compared to 25.3 % surgical complications for RC patients and 22.3 % medical complications [17]. Table 3 shows the number of patients with postoperative complications and their association with survival and recurrences, stratified for colon and rectal cancer.

**Table 3:** Proportion of complications and the association with overall survival (OS) and disease free period (DFP)

Complications		Colon cancer	Rectal cancer		*P* value	
Complications	Yes	315 (41.1)	134 (49.3)		*0.019*	
	No	452 (58.9)	138 (50.7)			
Association complications and overall survival (OS)						
		5-years OS	HR (95%CI)	*p* value	Adjusted^a^ HR (95%CI)	*p* value
Complications in	No	68.3 (62.9–73.0)	1.0 (ref)	*<0.001*	1.0 (ref)	*<0.001*
colon cancer patients	Yes	49.7 (43.4–55.7)	2.02 (1.59–2.55)		1.57 (1.23–2.01)	
Complications in	No	77.4 (69.7–84.0)	1.0 (ref)	*0.002*	1.0 (ref)	*0.015*
rectal cancer patients	Yes	56.6 (45.5–66.3)	2.04 (1.29–3.22)		1.79 (1.12–2.87)	
Association complications and disease free period (DFP)						
		5-years DFP	HR (95%CI)	*p* value	Adjusted^a^ HR (95%CI)	*p* value
Complications in	No	77.8 (73.3–81.6)	1.0 (ref)	0.1	1.0 (ref)	0.1
colon cancer patients	Yes	71.2 (64.8–76.7)	1.30 (0.95–1.79)		1.29 (0.93–1.80)	
Complications in	No	75.8 (67.2–82.5)	1.0 (ref)	0.2	1.0 (ref)	0.2
rectal cancer patients	Yes	64.4 (53.4–73.4)	1.36 (0.84–2.21)		1.43 (0.86–2.39)	

The table shows the cumulative proportion surviving 5 years after surgery, the cumulative proportion surviving disease free 5 years after surgery, the related hazard ratios and adjusted hazard ratios

The italicized items have a *p* - value smaller than 0.05, which is concidered statistically significant in our study

*OS* overall survival, *DFP* disease free period, *HR* hazard ratio, *Ref* reference category

^a^Adjusted for age, T-stage, grade and emergency setting

The 5-year overall survival for patients with complications with RC was higher than for CC patients, 56.6 % (45.5–66.3) versus 49.7 % (43.4–55.7), respectively. Differences in 5-year overall survival for patients with and without complications were significant for both CC and RC patients. The adjusted HR for CC patients was 1.57 (1.23–2.01; *p* < 0.001) and for RC the HR was 1.79 (1.12–2.87; *p* = 0.015).

The adjusted differences in the DFP between patients with and without complications showed a trend towards significance for both CC and RC patients with an adjusted HR of 1.29 (0.99–1.80; *p* = 0.1) and 1.43 (0.86–2.39; *p* = 0.2), respectively.

**Fig. 2:**
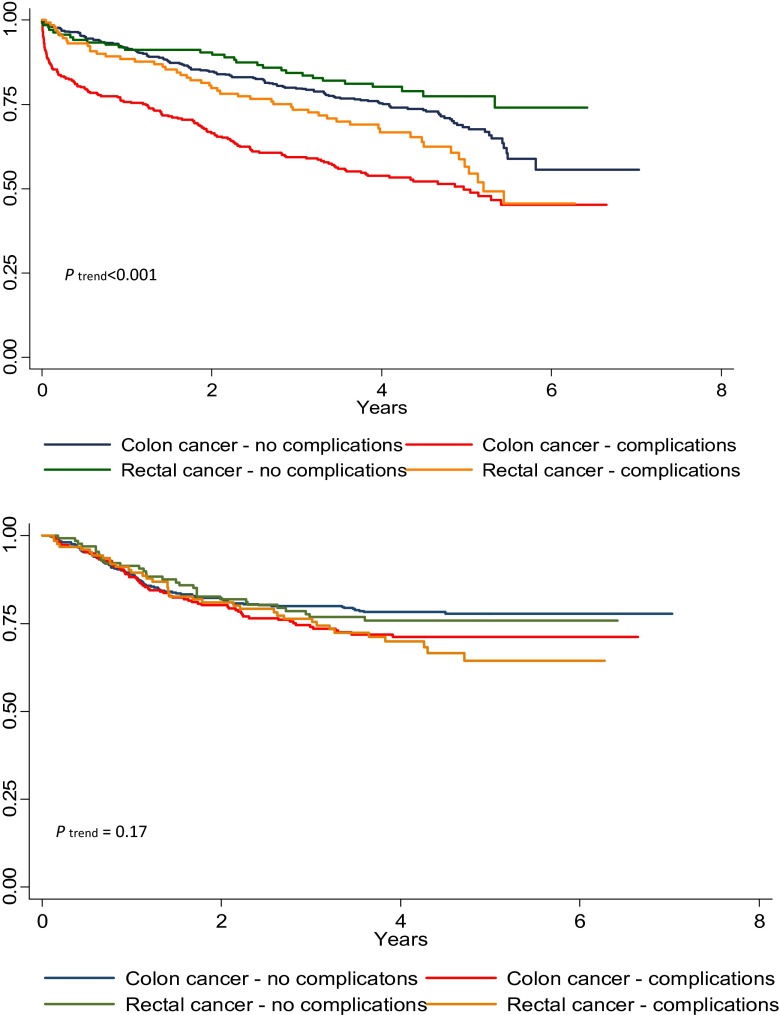
Survival curves of the overall survival (top graph) and disease free period (bottom graph) for colon and rectal cancer patients with and without complications

Figure 2 shows the overall survival and disease free period for CC and RC patients with and without complications. Differences in the OS between the patients with and without complications were statistically significant; *p* < 0.001 for CC and *p* = 0.002 for CR. There is also a significant difference in the overall survival between CC and RC patients with complications (*p* = 0.029), whilst the difference shows a trend towards significance between CC and RC patients without complications (*p* = 0.074). The figure shows a high mortality rate in the short-term postoperative period (30 days) for CC patients. The DFP curve shows a statistical trend towards a higher recurrence rate in the CC and RC groups with complications (*p* = 0.104 and 0.204 for CC patients or CR patients with and without complications, respectively).

## Discussion

In this study, a large difference has been observed in the short-term mortality of RC and CC patients with a higher mortality for patients with colon cancer. Patients with rectal cancer experience complications more often after surgery. Stratified by CC and RC, complications are associated with a worse overall survival, however not with recurrences. Patients with colon cancer and complications after surgery have the worst survival, compared to rectal cancer patients and patients without complications.

### Age and gender

The proportion of males was higher for RC than for CC. This can partly be explained by the age difference between RC and CC patients; RC patients are evenly distributed among the age groups, whilst many of the CC patients are older than 75 years. This unequal gender distribution at high age due to the difference in lifespan is also shown in other studies [12, 15]. This suggests CC to be a more age-related disease than RC and indicates the two malignancies must have different aetiologies [2–5, 18]. Old age is known to have a negative effect on the recovery of surgery for CC patients. Many studies found age to be a significant predictor of more complications and higher short-term mortality in colon and rectal cancer, more specific for medical complications but not for surgical complications [13, 19–22].

### Complications

Our analyses on complications revealed discrepancies in the prevalence and in the short-term effects of complications on patient survival between rectal cancer and colon cancer patients. Of all RC patients, 49.3 % experienced complications versus 41.1 % of CC patients. Reported percentages in literature of colorectal postoperative complications include 46.5 % by Biondo et al. (2004) and 23 % major complications by Al-refaie et al. (2011) [13, 19]. Iversen et al. (2008) noted a frequency of 27.8 % for surgical complications, and Marusch et al. (2005) 20 %, which are similar to our results [20, 21]. Law et al. (2007) and McArdle et al. (2005) also reported that medical complications are more common in CC patients whilst surgical complications are more common in RC patients [23, 24].

The effect of surgery and surgical complications on patient mortality is known to increase with age. Elderly are not only more prone to suffer from complications, they are also more likely to succumb to them [25, 26].

### Short-term mortality

The short-term survival of CC patients is worse than that of RC patients due to more excess mortality in the first few months after surgery. Short-term excess mortality rates of CC are 12.5 % versus a much lower 8.5 % for RC after 1 year. These findings are in line with the overall 1-year excess mortality rates of 10.9 % for CC patients versus 4.8 % for RC patients reported by Gooiker et al. (2012) [12]. The large difference visible in the first few months of the relative survival between RC and CC confirms this observation. Relative survival was used to correct for the overestimation of the effect of cancer on survival [15]. Crude survival reflects the mortality rates regardless of cause, whilst relative survival is defined as the ratio of observed versus expected mortality, which is valuable with large age differences within the patient population. Utilizing these figures to compute the relative excess risk also enables a more accurate comparison of the effects of surgery on RC and CC patients. The relative survival differences were borderline significant.

Complications had a negative effect on the overall survival of both rectal cancer and colon cancer patients. However, the short-term effects of complications were worse for CC patients, and accounts for the large differences in 1-year excess mortality between RC and CC. The discrepancy in the effect of complications on survival, together with the discrepancy in the prevalence of complications is described as the failure-to-rescue difference between colon- and rectal cancer complications [26]. The study by Henneman et al. (2014) demonstrates that factors such as age, ASA-status, comorbidity and colon- or rectum-resections are of significant influence on the failure-to-rescue in colorectal cancer, whilst factors such as TNM-stage, neo-adjuvant therapy or approach (laparoscopy/laparotomy) are not.

### Long-term survival and recurrences

The overall survival differences for patients with and without complications remained after adjusting for both RC and CC patients, with a worse survival rate for patients with complications. Complications in CC and RC patients result in a stable long-term increased mortality rate for as long as the follow-up was recorded in this study (up to 7 years). These results correspond with those found by Dekker et al. (2011) [15].

A statistical trend was found towards a worse disease free period in the CC and RC groups with complications. Systemic mechanisms such as the immune system, which has significant influence on the tumour progression for colorectal and breast-cancer, could explain both the occurrence of multiple complications and susceptibility to recurrences [27, 28]. Moreover, postoperative infectious complications could lead to an increase of systemic cytokines by the activated immune system. These cytokines, also associated with wound-healing, reduce anti-antigenic factors and act as growth factors, thus stimulating tumour-cell growth and proliferation of circulating micro-metastases [29]. Prolonged decreased mobility, associated with complications has also been revealed to affect intestinal transit time, levels of insulin-like growth factors and the immune system [30]. In stage III, CC complications lead to a delayed start of chemotherapy, which is associated with a worse disease free and overall survival [31]. More detailed analyses in future studies on the type of postoperative complications or histopathological analysis on the lymphocytic infiltration of the tumour tissue could provide more clarity on this matter. Such knowledge could improve the prognostic accuracy and might allow for patient specific selection for chemotherapy treatment.

### Limitations

Limitations inherent to the retrospective design apply to our study. The preoperative period of neoadjuvant chemo and/or radiotherapy, usually more frequent in RC, could form a selection period to surgery. Furthermore our study lacks detail on the impact of different types of complications as well as the effect of age on the incidence and response to complications. A more in-depth study on these topics could reveal more about the nature of the discrepancy in the frequency and effect of complications.

Patients with the primary tumour located in the rectosigmoid were excluded due to the uncertain nature of these malignancies. Crude analyses including this group indicated they are clinically more similar to RC patients than to CC patients, and classifying them as such enhanced the statistical differences between CC and RC.

Colon and rectal cancer patients have a different age distribution, and previous studies show the importance of age on survival and the risk for and impact of complications [13, 19–22, 25, 26]. However, no studies have been conducted which compare the effects of complications for different age groups and with colon and rectal cancer analysed separately. Such study would provide more insight into the effect of age on the development and severity of complications, which we have proven to be very different for colon and rectal cancer, and could also explain the difference in the failure-to-rescue.

The proportion of patients with more advanced stage (T4) and the proportion of patients treated in an emergency setting were larger for colon cancer patients. These factors are associated with complications, overall survival and recurrences. Although we adjusted for these factors in the multivariable analyses, some residual confounding might be present due to other factors associated with more advanced stage and emergency setting which we could not adjust for in the analyses. Grade was also distributed differently between colon and rectal cancer, however not associated with the outcome in the present study.

A population-based screening program was initiated in the Netherlands for both males and females in the age of 55 to 75 years old, which will likely increase the incidence of low stage CC. This might results in a change in the discrepancies between CC and RC patients. However, it is too early to estimate these changes.

### Conclusion

This study enucleates some of the epidemiological differences and outcomes of colon and rectal cancer surgery which are of clinically relevant prognostic value. Short-term mortality is higher for patients with colon cancer, especially those who experience complications, although this was not associated with recurrences. Detailed analyses of factors leading to and prevention of complications should be the focus to improve the short-term outcomes of patients with colon cancer.

## References

[1] Iacopetta B (2002). Are there two sides to colorectal cancer?. International journal of cancer Journal international du cancer.

[2] Wei EK, Giovannucci E, Wu K, Rosner B, Fuchs CS, Willett WC (2004). Comparison of risk factors for colon and rectal cancer. International journal of cancer Journal international du cancer.

[3] Terry PD, Miller AB, Rohan TE (2002). Obesity and colorectal cancer risk in women. Gut.

[4] Colditz GA, Cannuscio CC, Frazier AL (1997). Physical activity and reduced risk of colon cancer: implications for prevention. Cancer Causes Control.

[5] Kapiteijn E, Liefers GJ, Los LC, Kranenbarg EK, Hermans J, Tollenaar RA (2001). Mechanisms of oncogenesis in colon versus rectal cancer. J Pathol.

[6] Li FY, Lai MD (2009). Colorectal cancer, one entity or three. J Zhejiang Univ Sci B.

[7] Kornmann M, Staib L, Wiegel T, Kron M, Henne-Bruns D, Link KH (2013). Long-term results of 2 adjuvant trials reveal differences in chemosensitivity and the pattern of metastases between colon cancer and rectal cancer. Clin Colorectal Cancer.

[8] Guyot F, Faivre J, Manfredi S, Meny B, Bonithon-Kopp C, Bouvier AM (2005). Time trends in the treatment and survival of recurrences from colorectal cancer. Annals of oncology: official journal of the European Society for Medical Oncology / ESMO.

[9] Elsaleh H, Joseph D, Grieu F, Zeps N, Spry N, Iacopetta B (2000). Association of tumour site and sex with survival benefit from adjuvant chemotherapy in colorectal cancer. Lancet.

[10] Iacopetta B, Kawakami K, Watanabe T (2008). Predicting clinical outcome of 5-fluorouracil-based chemotherapy for colon cancer patients: is the CpG island methylator phenotype the 5-fluorouracil-responsive subgroup?. Int J Clin Oncol.

[11] Willett CG, Tepper JE, Shellito PC, Wood WC (1989). Indications for adjuvant radiotherapy in extrapelvic colonic carcinoma. Oncology (Williston Park).

[12] Gooiker GA, Dekker JW, Bastiaannet E, van der Geest LG, Merkus JW, van de Velde CJ (2012). Risk factors for excess mortality in the first year after curative surgery for colorectal cancer. Ann Surg Oncol.

[13] Al-Refaie WB, Parsons HM, Habermann EB, Kwaan M, Spencer MP, Henderson WG (2011). Operative outcomes beyond 30-day mortality: colorectal cancer surgery in oldest old. Ann Surg.

[14] Paun BC, Cassie S, MacLean AR, Dixon E, Buie WD (2010). Postoperative complications following surgery for rectal cancer. Ann Surg.

[15] Dekker JW, van den Broek CB, Bastiaannet E, van de Geest LG, Tollenaar RA, Liefers GJ (2011). Importance of the first postoperative year in the prognosis of elderly colorectal cancer patients. Ann Surg Oncol.

[16] Oncoline. Colorectal carcinoma. In: Oncoline, editor. Guidelines oncological care. 3.0 ed: Intergral Cancercentre Netherlands (IKNL); 2014.

[17] Breugom AJ, van Dongen DT, Bastiaannet E, Dekker FW, van der Geest LG, Liefers GJ, et al. Association between the most frequent complications after surgery for stage I-III colon cancer and short-term survival, long-term survival, and recurrences. Ann Surg Oncol 2016. 10.1245/s10434-016-5226-z 27075325

[18] Servomaa K, Kiuru A, Kosma VM, Hirvikoski P, Rytomaa T (2000). p53 and K-ras gene mutations in carcinoma of the rectum among Finnish women. Mol Pathol.

[19] Biondo S, Pares D, Frago R, Marti-Rague J, Kreisler E, De Oca J, et al. (2004) Large bowel obstruction: predictive factors for postoperative mortality. Dis *Colon rectum* 47:1889–1897 10.1007/s10350-004-0688-7 15622582

[20] Iversen LH, Bulow S, Christensen IJ, Laurberg S, Harling H, Danish Colorectal Cancer G (2008). Postoperative medical complications are the main cause of early death after emergency surgery for colonic cancer. Br J Surg.

[21] Marusch F, Koch A, Schmidt U, Steinert R, Ueberrueck T, Bittner R (2005). The impact of the risk factor "age" on the early postoperative results of surgery for colorectal carcinoma and its significance for perioperative management. World J Surg.

[22] Khan MR, Bari H, Zafar SN, Raza SA (2011). Impact of age on outcome after colorectal cancer surgery in the elderly—a developing country perspective. BMC Surg.

[23] Law WL, Choi HK, Lee YM, Ho JW, Seto CL (2007). Anastomotic leakage is associated with poor long-term outcome in patients after curative colorectal resection for malignancy. J Gastrointest Surg.

[24] McArdle CS, McMillan DC, Hole DJ (2005). Impact of anastomotic leakage on long-term survival of patients undergoing curative resection for colorectal cancer. Br J Surg.

[25] Dekker JW, Gooiker GA, Bastiaannet E, van den Broek CB, van der Geest LG, van de Velde CJ (2014). Cause of death the first year after curative colorectal cancer surgery; a prolonged impact of the surgery in elderly colorectal cancer patients. Eur J Surg Oncol.

[26] Henneman D, Ten Berge MG, Snijders HS, van Leersum NJ, Fiocco M, Wiggers T (2014). Safety of elective colorectal cancer surgery: non-surgical complications and colectomies are targets for quality improvement. J Surg Oncol.

[27] Menon AG, Janssen-van Rhijn CM, Morreau H, Putter H, Tollenaar RA, van de Velde CJ (2004). Immune system and prognosis in colorectal cancer: a detailed immunohistochemical analysis. Laboratory investigation; a journal of technical methods and pathology.

[28] Gujam FJ, Edwards J, Mohammed ZM, Going JJ, McMillan DC (2014). The relationship between the tumour stroma percentage, clinicopathological characteristics and outcome in patients with operable ductal breast cancer. Br J Cancer.

[29] Neeman E, Ben-Eliyahu S (2013). Surgery and stress promote cancer metastasis: new outlooks on perioperative mediating mechanisms and immune involvement. Brain Behav Immun.

[30] Wolin KY, Yan Y, Colditz GA, Lee IM (2009). Physical activity and colon cancer prevention: a meta-analysis. Br J Cancer.

[31] Hendren S, Birkmeyer JD, Yin H, Banerjee M, Sonnenday C, Morris AM (2010) Surgical complications are associated with omission of chemotherapy for stage III colorectal cancer. Dis *Colon rectum* 53:1587–1593 10.1007/DCR.0b013e3181f2f202 21178851

